# Incidence of back pain from initial presentation to 3 years of follow-up in subjects with untreated adolescent idiopathic scoliosis

**DOI:** 10.1007/s43390-023-00794-8

**Published:** 2023-11-28

**Authors:** Kenney Ki Lee Lau, Kenny Yat Hong Kwan, Jason Pui Yin Cheung, Janus Siu Him Wong, Graham Ka Hon Shea, Karlen Ka Pui Law, Kenneth Man Chee Cheung

**Affiliations:** 1 Department of Orthopaedics and Traumatology, School of Clinical Medicine, Li Ka Shing Faculty of Medicine, The University of Hong Kong, Pokfulam, Hong Kong; 2 Department of Orthopaedics and Traumatology, The University of Hong Kong Shenzhen Hospital, Shenzhen, China

**Keywords:** Adolescent idiopathic scoliosis, Back pain, Natural history, Incidence, Conservative treatment

## Abstract

**Background:**

Although back pain may be present in subjects with adolescent idiopathic scoliosis (AIS), its natural history is unknown. Therefore, this study evaluated the incidence of back pain in scoliotic adolescents longitudinally.

**Methods:**

This retrospective analysis examined prospectively collected pain subscale data of the Scoliosis Research Society questionnaire between the initial presentation and up to 3 years of follow-up. Consecutive subjects with AIS aged 10–18 at baseline managed by observation within the study period were included. Study subjects with at least one time point of follow-up data were considered. Alternatively, a group with physiotherapy-treated was also included for comparison.

**Results:**

We enrolled 428 subjects under observation. The incidence of back pain among study subjects was 14.7%, 18.8%, and 19.0% for the first year, second year, and third year of follow-up, respectively. Most experienced mild pain (1 out of 5 points) throughout the study. Neither incidence nor intensity of pain significantly differed between subjects under observation and received physiotherapy. Additionally, study subjects with a new onset of back pain had poorer function, self-image, and mental health scores than those without pain.

**Conclusion:**

We investigated the incidence of back pain longitudinally in subjects suffering from AIS. Further validation of the current results is warranted.

## Introduction

Scoliosis is the prevalent form of spinal deformity affecting about 3% of the population [1]. Approximately 85% are diagnosed with adolescent idiopathic scoliosis (AIS) which is found during adolescence without a specific underlying cause [2]. Scoliotic adolescents with mild to moderate curvatures are often asymptomatic, except some of them may experience pain over the spine [3]. It has been shown that individuals with AIS possess an elevated risk of developing back pain compared to the general population of the same age [4]. Given the coronal deformity is a permanent condition in most cases (i.e. the curve is still maintained following conservative treatments), the population of scoliosis are exposed to back pain throughout their lifespan. Consequently, those with AIS and back pain together may develop poor mental health or even psychological distress [5].

Nevertheless, back pain associated with AIS may be neglected in clinical practice because the pain intensity may not be considered clinically relevant [6]. Although children with AIS may only experience mild back pain [7, 8], some studies proposed that having pain episodes during adolescence resulted in these same individuals being prone to suffer from the pain again in adulthood [9, 10]. Our recent study also suggested that the severity of painful young adults treated conservatively was as significant as 7 out of 10 on a numeric rating scale [11]. Therefore, it is crucial to characterise and prognosticate back pain within the context of AIS.

In the literature, the prevalence of back pain has been studied in the AIS population but has never been investigated longitudinally [12–14]. The natural history of back pain is lacking. To address the knowledge gaps mentioned, we aimed to evaluate the incidence of back pain dating from the first consultation to three years of follow-up in subjects with AIS.

## Materials and methods

This manuscript was written according to the STrengthening the Reporting of OBservational studies in Epidemiology (STROBE) statement [15].

### Study design

The present project was a retrospective analysis of prospectively collected clinical data implemented in a single academic medical centre. This study complied with the World Medical Association Declaration of Helsinki [16], and was approved by the local institutional review board (reference number: UW 22-257).

### Setting

The study site is a tertiary referral centre for scoliosis and is one of the only two designated local hospitals specialising in the management of spinal deformities. Apart from the routine radiographic assessment, we have embedded the revised 22-item Scoliosis Research Society questionnaire (SRS-22r) as the patient-reported outcome measure during clinic visits [17]. Due to the voluntary basis, discrete drop out at any time point was expected. We retrieved the archives of potential candidates at their initial presentation, as well as follow-ups of 1 year, 2 years, and 3 years. Data collection was carried out over 16 years, from Feb 2006 to Jan 2023.

### Participants

We included subjects with a diagnosis of AIS aged 10–18 at baseline. Those under the management of observation (i.e. no active intervention received) within the study period were eligible for inclusion. Candidates were required to have at least 1 year of follow-up data. For subjects with bracing and surgery, we extracted only the data still within the observation phase without any treatment offered. Conversely, some subjects may be referred to physiotherapy services for pain relief. As such, we have comprised them as a subgroup analysis for comparison with the study group. The exclusion criteria were as shown below, including (I) spinal trauma, injury, fracture, or tumour, (II) congenital spine abnormalities, and (III) history of bracing or surgery for scoliosis. Study subjects who missed the second and/or third year of follow-up data were still counted for their remaining available data (i.e. missing second year only, missing third year only, and missing both years), yet they were eliminated from calculation at the time point with missing data. Eligibility was affirmed by reviewing the electronic medical records.

### Measurement

The variable of interest was measured by the SRS-22r. It is a well-established, scoliosis-specific, patient-based outcome measure [18, 19]. There are four domains estimated for the condition of the back [20], comprising function, pain, self-image, and mental health. Each domain is evaluated by five questions on a 5-point scale. A score of five is the best, and one is the worst. In addition, we employed the posteroanterior standing view of the whole spine or whole body for the radiographic assessment. Curve magnitude was assessed digitally using the method described by Cobb on a picture archiving and communication system (PACS) software.

### Variables

**Fig. 1:**
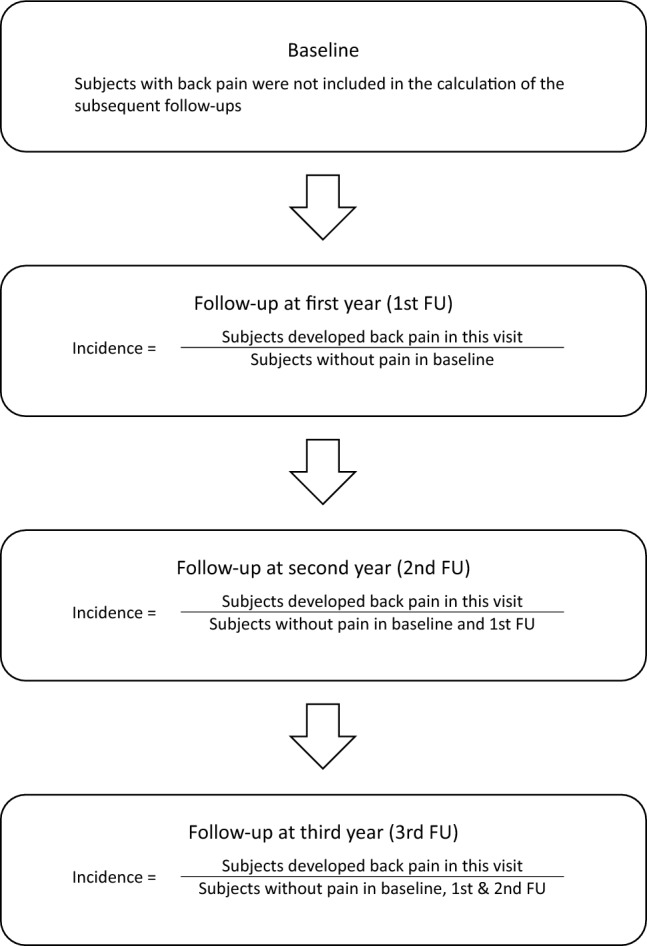
Schematic flow on the calculation of the incidence

The presence of back pain was determined by question one and two of the SRS-22r, namely “which one of the following best describes the amount of pain you have experienced during the past 6 months?” and “which one of the following best describes the amount of pain you have experienced over the last month?”. Answers other than “none” were defined as having back pain. Further, the intensity of pain was classified into four levels, involving mild, moderate, moderate to severe, and severe. In particular, the incidence of back pain was only calculated in the follow-ups. For calculating the new onset of back pain at each time point (see Fig. 1), subjects with pain-free in previous time point(s) were accounted for the denominator (i.e. justified by the first question of SRS-22r). The occurrence of pain was based on the second question of SRS-22r. Moreover, the severity of the spinal curvature was measured in terms of the Cobb angle for the major structural curve [21]. Demographic data of age at initial presentation and gender was also assembled.

### Statistical analysis

Descriptive statistics were reported for the incidence and intensity of back pain. The Chi-square test was performed to compare subjects under observation and physiotherapy. The independent samples *t*-test was performed between study subjects with and without back pain. Mean difference (MD) and 95% confidence interval (CI) were supplemented for between-group comparisons. All data were analysed through the statistical package for the social sciences (SPSS) software version 28.0 and the *p* < 0.05 was set as the level of significance.

## Results

**Fig. 2:**
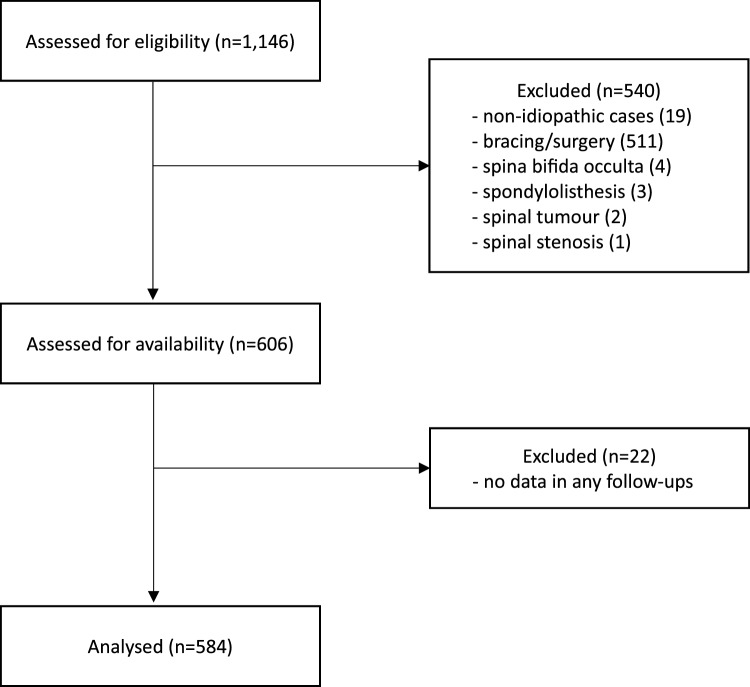
Flow diagram of participants’ selection

A total of 1146 candidates were screened for eligibility (see Fig. 2). Five hundred and forty individuals were excluded due to not meeting the inclusion criteria (*n* = 530) or having spinal problems (*n* = 10). The remaining 606 subjects were sought for retrieval, and 22 persons were further excluded because of the unavailability of the longitudinal data. Subsequently, we included 584 participants in the present study. Of them, 428 were under observation and 156 had received physiotherapy (see Table 1). Demographics of the study group were displayed as follows, mean age of 13.6 ± 1.6 years and average major Cobb angle of 21° ± 6° at initial presentation, and 69% were females.

**Table 1:** Characteristics of participants

	Observation	Physiotherapy
Sample size	428	156
Gender	69% females	78% females
Age at initial presentation	13.6 ± 1.6 years	13.1 ± 1.6 years
Major Cobb angle at initial presentation	21° ± 6°	24° ± 7°
Sessions attended	–	4 ± 4 visits

**Fig. 3:**
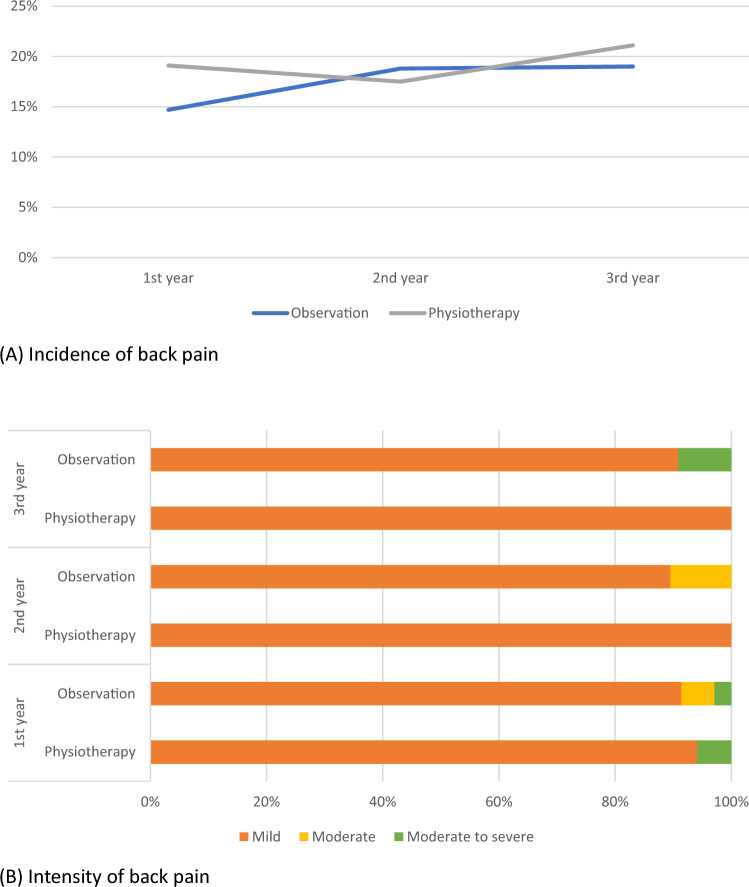
Back pain between subjects under observation and physiotherapy

In essence, the incidence of back pain amongst subjects with untreated AIS was 14.7% (*n* = 35/328), 18.8% (*n* = 19/101), and 19.0% (*n* = 11/58) in the first, second, and third year of follow-up, respectively. Approximately 90% had only mild pain intensity throughout the study period, and only a small proportion had moderate and moderate to severe pain. By comparing subjects under observation with those who underwent physiotherapy, there were no statistically significant difference in the incidence and intensity of back pain between groups (see Fig. 3).

**Fig. 4:**
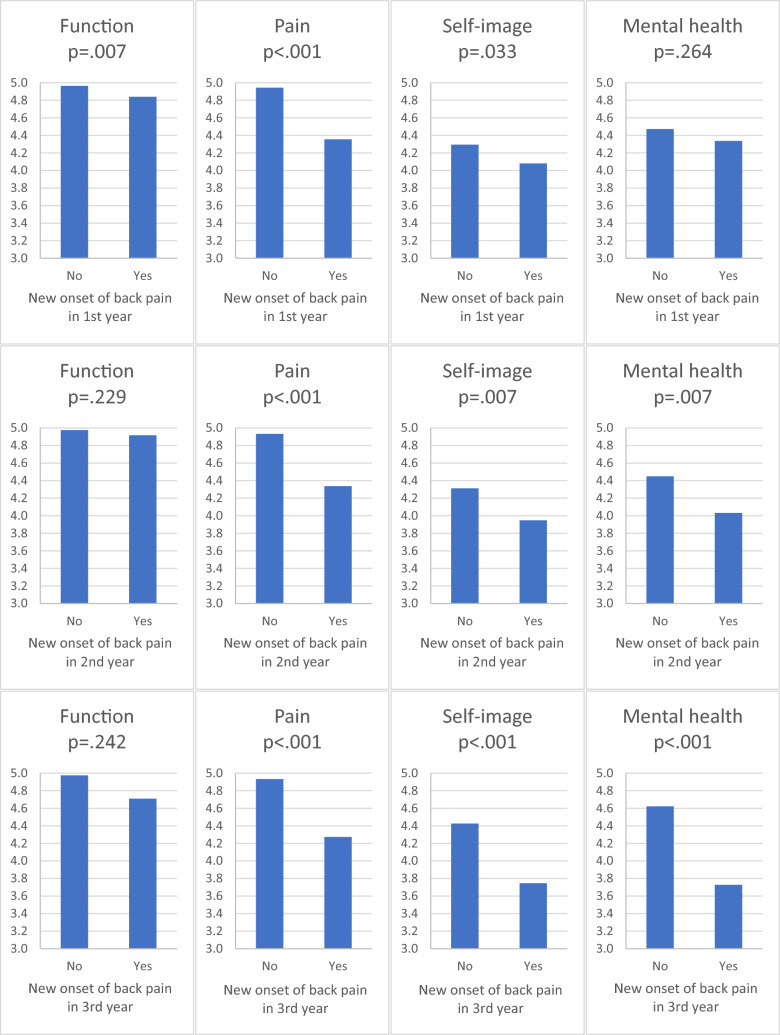
SRS-22r scores in study subjects with and without back pain

As for the patient-reported outcome measures (see Fig. 4), study subjects with new onset of back pain had inferior SRS-22r scores compared to those without pain in the first year (function: MD = 0.12, CI = 0.21 and 0.04, *p* = 0.007; pain: MD = 0.59, CI = 0.66 and 0.52, *p* < 0.001; self-image: MD = 0.21, CI = 0.41 and 0.02, *p* = 0.033; and mental health: MD = 0.13, CI = 0.38 and 0.06, *p* = 0.264), the second year (function: MD = 0.06, CI = 0.16 and 0.04, *p* = 0.229; pain: MD = 0.59, CI = 0.67 and 0.52, *p* < 0.001; self-image: MD = 0.36, CI = 0.63 and 0.10, *p* = 0.007; and mental health: MD = 0.42, CI = 0.72 and 0.12, *p* = 0.007), and the third year of follow-ups (function: MD = 0.27, CI = 0.74 and 0.21, *p* = 0.242; pain: MD = 0.66, CI = 0.94 and 0.38, *p* < 0.001; self-image: MD = 0.68, CI = 1.1 and 0.29, *p* < 0.001; and mental health: MD = 0.89, CI = 1.2 and 0.55, *p* < 0.001).

**Fig. 5:**
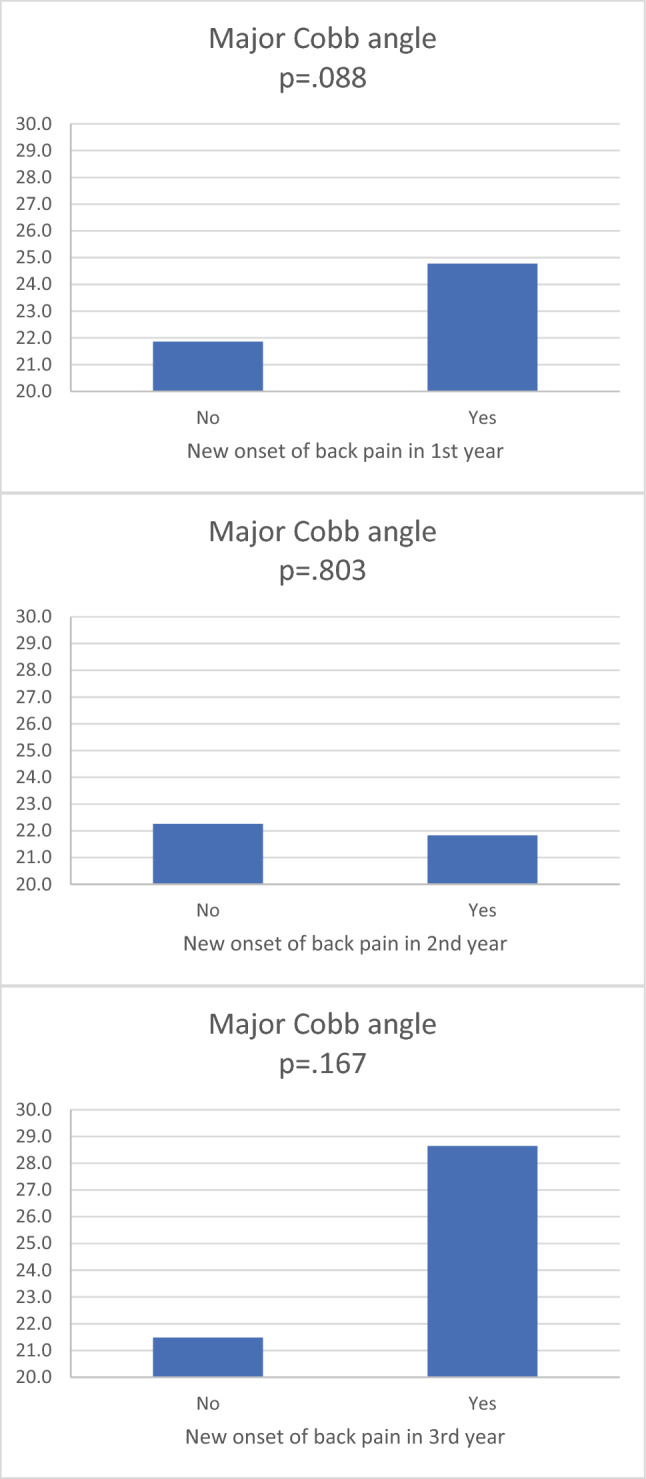
Major Cobb angle in study subjects with and without back pain

Additionally, major Cobb angles were larger in study subjects with pain than their counterparts in the first year (MD = 2.91°, CI = 0.45° and 6.28°, *p* = 0.088), the second year (MD = 0.43°, CI = 3.82° and − 2.97°, p = 0.803), and the third year of follow-ups (MD = 7.16°, CI = 3.51° and 17.83°, *p* = 0.167), despite statistical significances were not achieved (see Fig. 5).

## Discussion

This was the first study to investigate the natural history of back pain in subjects with AIS. We found that the incidence of back pain was about 15–19% annually among those under observation. Unexpectedly, treatment by physiotherapy did not reduce the occurrence of pain. Even though our cohort only experienced mild pain within the first 3 years since the initial presentation, their long-term outcomes into adulthood were still of concern.

We have evaluated the incidence instead of the prevalence of back pain in scoliotic adolescents. This work has never been accomplished until now. Based on the statistics from this study, we hypothesised that the number of people with AIS suffering from new onset of back pain in the future could grow if left untreated. This advocate is indirectly sustained by the literature. During adolescence, the prevalence of back pain began at a 9% rate in the population with AIS [12]. The figure progressed along with the age. A similar cohort of young adults demonstrated a prevalence of 20% with pain [11]. Furthermore, middle age adults with idiopathic scoliosis had reached a 69% prevalence [22]. Turning to the elderly, 77% of scoliotic subjects were tolerated with back pain [23]. All these numbers were significantly higher than in the global population with pain [24]. In consequence, we speculated that the burden of back pain in AIS is significant.

Notably, the distribution of back pain between subjects with AIS and the paediatric population with no scoliosis is yet to be known. Results from the studies in Greece and Brazil have illustrated the prevalence and incidence of low back pain in children (aged 7–14) and adolescents (aged 14–18) were about 22% and 19% [25, 26], respectively. In contrast, data from systematic reviews and a meta-analysis have revealed lower rates [27, 28]. These numbers seemed inferior to the current results and the literature on scoliosis. Nonetheless, the comparison was slightly different between the two groups in terms of the definition of pain. Back pain in AIS was referred to as the pain in either thoracic and/or lumbar, but it was generally considered only the pain in the lower back among the general population. We suspect the variations were partly due to the comparability.

Although pain intensity was counted as mild in adolescents that may be clinically irrelevant, we may underestimate the consequences of back pain. Its circumstances in adolescence could lead to severe back problems in older ages [29–32], which may necessitate surgical intervention. A recent study from our team has presented a seriously high severity of pain in adulthood (7 on an 11-level numeric rating scale) [11]. Grauers et al. [22] described that 29% and 37% of adults with back pain had compromised their activity level and had recurrent daily pain. The longest follow-up study from Weinstein et al. [23] declared that 32% of the elderly had unbearable back pain (5 out of 5 points), and 91% had it for more than two years. We can foresee that the problem of pain would be aggravated as ageing and becoming inevitable. Therefore, clinicians should manage back pain when subjects are still young (e.g. psychosocial intervention may be adopted) and prevent progression into a more frequent and clinically relevant pain situation [33–35].

Originally, we expected that those undergoing physiotherapy would have a lower incidence of back pain no matter any forms of treatment, but the current results did not support this assumption. It may be related to the indication for the referral of physiotherapy service as it was variable. While subjects with AIS are usually referred for physiotherapeutic scoliosis-specific exercises [36], some may be referred for general range of motion and stretching exercises [37]. There may also be differences in particular subjects who received more lengthy outpatient treatments, whereas others are taught exercises to do themselves at home after a limited number of training sessions. As such, the effectiveness of each physiotherapy modality on back pain in AIS was not known.

Regarding the influences of back pain, we noticed that the pain, function, and mental health scores have exceeded the minimal clinically important difference [38]. The perception of pain is not just a functional impairment but also has a negative psychological effect [39]. This vicious circle of back pain begins with the development of back pain leading to poorer mental health [11], and a reduced mental state could be susceptible to perceiving the pain thus decreasing the threshold of pain sensation [40]. Provided untreated subjects would not have a straight spine in their life, we need to resolve the contributing factors (back pain) for the worsened quality of life.

We noted some limitations in the present study related to the methodological quality. For the outcome measures at baseline, the condition of pain-free was operationally defined as having no history of pain episodes over the past six months. Notwithstanding the chance is rare that subjects may have had pain in their lifetime, but not recurrent prior to the initial presentation. Owing to the retrospective nature of the study design, it is uncertain whether the SRS-22r questionnaire reflects lifetime prevalence or prevalence around the time of the survey. The same question was used to exclude subjects with back pain episodes from the incidence calculation. Similarly, the period did not fully cover the intermission between the two time points. We also noticed that the profile of back pain was not comprehensively assessed as the measurement used was not the most appropriate. In spite of some cases of specific back pain (i.e. spina bifida occulta, spondylolisthesis, spinal tumour, and spinal stenosis) having been removed, other factors like intervertebral disc degeneration and facet joint degeneration were not evaluated. The current results should be interpreted with caution.

Future investigations should aim at the natural history of back pain in subjects with AIS following a lengthier period so that we can better understand its prognosis. Besides, a better understanding of the pain is warranted on how various factors interplay in scoliosis. Comparative analysis of the incidence and prevalence of back pain is desired between subjects with and without AIS. Whilst the effects of bracing and surgery on the development of back pain remain controversial, higher level of evidence studies should address this issue. Proactive intervention should also be studied to avoid back pain in subjects with AIS.

## Conclusion

We were the first team to attempt to study the natural history of back pain in terms of incidence within the population with untreated AIS. The incidence was about 15–19% annually, accompanied by mild pain intensity and poor mental health. Physiotherapy did not result in a definite improvement in pain. Spine surgeons may undervalue the implications of back pain in AIS. Therefore, consultation with patients regarding the management of pain is needed, which could include therapist-led prevention programmes. Further validation of the current results is required for a longer follow-up duration in a multi-centre setting.
